# Comparatively Analyzing the Impact of Government Subsidy and Carbon Tax Policy on Authorized Remanufacturing

**DOI:** 10.3390/ijerph18168293

**Published:** 2021-08-05

**Authors:** Biao Li, Yong Geng, Xiqiang Xia, Dan Qiao, Hao Wang

**Affiliations:** 1 College of Business, Zhengzhou University, Zhengzhou 450001, China; lib0023@zzu.edu.cn (B.L.); qiao0205@126.com (D.Q.); 2 College of Environmental Science and Engineering, Shanghai Jiao Tong University, Shanghai 200240, China; ygeng@sjtu.edu.cn; 3 Department of Geography and Planning, University of Toronto, Toronto, ON M5S 3G3, Canada; haow.wang@mail.utoronto.ca

**Keywords:** government subsidies, carbon tax, authorization remanufacturing, game model

## Abstract

Authorized remanufacturing is an important means to achieve green manufacturing and carbon neutrality. In this study, a game theory model between a manufacturer and a remanufacturer was constructed to analyze the impact of government subsidies and carbon tax policies on authorized remanufacturing. Based on the game theory model, the effects of two government policies on the optimal solution, namely, the unit cost of remanufacturing product authorization and the waste product recovery rate, were compared and analyzed. This analysis could provide a reference for the government to improve and formulate relevant remanufacturing policies. The main results are as follows: government subsidy policies may increase the unit cost of remanufacturing product authorization and the rates of waste product recovery; government carbon tax policies may not affect the unit cost of remanufacturing product authorization, and increase the rates of waste product recovery; the government subsidy policy may not affect the unit retail price of new products, and reduces the unit retail price of remanufactured products; the government subsidy and carbon tax policies may reduce sales of new products and increase sales of remanufactured products; the government subsidies may increase the revenue of the original equipment manufacturer (OEM) and the remanufacturer; and the government carbon tax policies may increase the revenue of the remanufacturer. However, government carbon tax policies increase the revenue of the OEM only when the new product carbon tax amount is higher than a certain threshold. The impact of the two policies on the environment is related to the ratio of the two products’ impact on the environment, i.e., the quota ratio between the unit government carbon tax of the new product and the unit government subsidy of the remanufactured product. Finally, the consumer surplus is maximized when the government adopts the subsidy policy and lowest when the government adopts the carbon tax policy.

## 1. Introduction

Reaching peak carbon emissions and carbon neutrality, and actively realizing a low-carbon economy, have become shared goals of all countries [1,2,3]. Currently, carbon emissions are mainly produced by the initial production process, particularly in energy-intensive industries such as steel and cement, the energy utilization of wastes such as straw and forestry waste, and the recycling of power batteries [4,5]. Among these, the inefficient recycling of waste products is a major challenge and creates carbon emissions. If the waste product is not handled correctly, it will not only have a negative impact on the environment, but also waste a large quantity of resources and energy [6]. Taking e-waste as an example, the *Global E-Waste Monitoring Report 2020* showed that, since 2014, the total amount of global e-waste has steadily increased, and about 53.6 million tons of e-waste was generated in 2019. Resource waste and the associated environmental pollution have become global problems, and have thus attracted significant attention from governments. Governments have sought development strategies to achieve sustainable economic development, and thus promote resource conservation and alleviate the negative impact of waste products on the environment [7,8]. The world’s three major economies—China, the United States, and the European Union—are the main forces in the global development of renewable energy, reducing the use of fossil energy, increasing the electrification rate, and reducing carbon dioxide emissions.

Remanufacturing is one of the key paths to achieve green manufacturing, carbon neutrality, and sustainable development [9]. Remanufacturing can maximize the value of manufacturing products, improve resource utilization, and significantly reduce the negative impact of waste products and other materials on the ecological environment and individual health [10,11,12,13]. The remanufacturing model mainly comprises remanufacturing by original equipment manufacturers and third parties, among which third-party remanufacturing is the mainstream model [14]. Third-party remanufacturing is divided into outsourced remanufacturing and authorized remanufacturing [15]. The choice of a third party for remanufacturing by original manufacturers based on intellectual property protection is an effective means for original manufacturers to participate in the remanufacturing process and obtain remanufacturing revenue [16,17,18]. In particular, when the per unit new product production cost is high or consumers perceive that the remanufactured product has a low value, authorized remanufacturing is an effective model [15]. However, original manufacturers lack the expertise that is required for remanufacturing and the recycling channels for waste products, and the benefits of remanufacturing are relatively small compared to those accruing from the production of new products. Original manufacturers are generally unwilling to engage in remanufacturing but, instead, rely on intellectual property rights. Because third-party remanufacturing involves issues such as the reputation of the OEM’s product, technology patents, and consumer market seizures, OEM authorization is often required to remanufacture waste products. Although patent infringement has a recycling effect and a positive impact on the environment, the courts in the United States and Japan have not tolerated this behavior [19]. Third parties engaged in remanufacturing often purchase 3PR licenses from OEMs and pay patent licensing fees [18]. 

Due to limited public awareness, the market share of remanufactured products is still relatively low [20,21]. With the improvements in the outsourcing and intellectual property systems, in addition to the realization of the international goals of carbon peaking and carbon neutrality, the proportion of remanufactured products, particularly authorized remanufactured products, will further increase in the future. This development will be important for green manufacturing and carbon emission reduction. However, because remanufacturing is based on the recycling and reuse of manufactured products, remanufactured products have a cost advantage and pose a competitive threat to manufactured products. This is also one of the important reasons for the relatively slow development of remanufactured products. 

One of the aims of the promotion of international cooperation through both market and non-market means is to also promote the implementation of emission reduction targets by all parties [22,23]. Although governments have formulated a series of policies and regulations to promote the development of the remanufacturing industry, the laws and regulations do not allow the infringement of the OEM’s intellectual property rights, despite the environmental benefits and resource protection provided by remanufacturing. According to the legislation associated with the property rights, the remanufacturer must obtain the authorization of the OEM for remanufacturing [19]. To encourage the development of remanufactured products, governments of various countries are making significant efforts to introduce various policies to directly or indirectly encourage the development of remanufactured products. Among these, direct encouragement policies mainly include government subsidies [24,25] and return policies [26,27]. Negative restrictive policies mainly include carbon taxes [28], carbon regulation [20], cap-and-trade schemes [29], and carbon permit allocation [30].

A strategy involving subsidies is one of the most effective means to promote the development of authorized remanufacturing enterprises [31,32,33,34,35]. A subsidy strategy aims to improve low-carbon enterprises’ R&D level [36], increase remanufacturing activities [37,38], and influence consumer preferences [39]. Government subsidies stimulate the demand for energy and the profit of energy service companies [40]. Various subsidies can be applied at different stages of industrial development, and mixed-subsidy policies have a greater impact on the number of remanufacturers and the overall quantity of remanufacture products. However, the literature has rarely analyzed the impact of government subsidies on the environment and social surplus [41], or examined the differences in the strategies of subsidizing the OEMs, the IRs, or both simultaneously. Previous research has only noted that when the government subsidizes the OEMs, the profits of the OEMs and the IRs will increase at the same time [42].

A carbon tax is also an effective means to effectively promote authorized remanufacturing [43]. The effective formulation and implementation of carbon tax rates can reduce carbon emissions in the remanufacturing supply chain [44,45]. Carbon tax policies are more conducive to carbon reduction than direct carbon control policies [30]. Due to the different characteristics of different remanufacturing companies, the government should set carbon tax rates based on the tax collection cycles and carbon tax effects to more effectively reduce carbon emissions [46].

To date, scholars have compared the impact of different government policies on carbon emission reduction and remanufactured product production. For example, previous research has compared and analyzed the impact of subsidy strategies and carbon regulation [20], carbon taxes and cap-and-trade schemes [29], carbon taxes and take-back legislation [47,48], and take-back and carbon emission capacity regulations [27]. 

At present, policy measures are focused on increasing carbon taxes and implementing carbon emission reduction subsidies. These two government policies have different effects on the development of the remanufacturing industry. Scholars generally believe that subsidy policies are more effective than carbon tax policies in curbing carbon emissions. This may be because, although remanufacturing subsidies promote firms’ profit, carbon regulation reduces profits [49]. Carbon taxes have a significant impact on pricing strategies, whereas subsidy strategies are beneficial to remanufactured products and manufacturers [50]. 

The current research generally uses the independent remanufacturer model for analysis. In addition, the research has not considered the impact of intellectual property protection costs on manufacturers and remanufacturers. Therefore, it is necessary to compare and analyze the effects of the two government policies on authorized remanufacturing. This analysis could provide a reference for governments to improve and formulate relevant remanufacturing policies. The current study compared and analyzed the impact of the two government policies on authorized remanufacturing. Based on the existing research, this study intended to address the three following issues:

1. What are the impacts of government subsidies on the unit retail price, demand, revenue, environment, and consumer surplus of manufacturing/remanufacturing?
2. What are the impacts of government carbon taxes on the unit retail price, demand, revenue, environment, and consumer surplus of manufacturing/remanufacturing?
3. Under what conditions can the government adopt subsidies or carbon tax policies to effectively promote the development of the remanufacturing industry and reduce the adverse effects of manufacturing/remanufacturing on the environment?

The structure of this article is as follows. Section 2 presents the game theory model. Section 3 provides the specific model construction and analysis results. Based on the game theory model, the effects of the two government policies on the optimal solution, such as the unit cost of remanufacturing product authorization and the waste product recovery rate, are compared and analyzed. Section 4 uses a well-known engine remanufacturer in China as an example to perform mathematical analysis and draw inferences. Section 5 discusses the research conclusions and outlook.

## 2. Model Formulation

### 2.1. Problem Description

**Figure 1:**
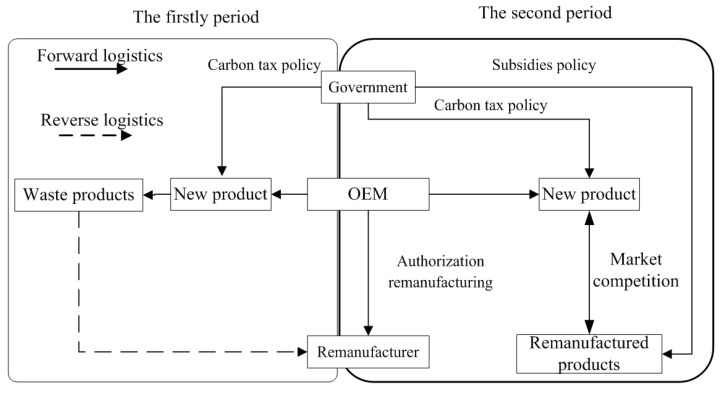
The game theory model of authorized remanufacturing based on government subsidies and carbon tax policies.

Two periods are considered in this study. In the first period, there are only new products in the market, and the decision variable is the retail price of the new product per unit. In the second period, under the protection of intellectual property rights, the OEM authorizes a remanufacturer to produce the former’s products. As a premise, authorization fees are charged by the OEM on the remanufacturer. The decision variable in the period is the amount of the authorization fee and the retail price of the new product per unit. Moreover, before remanufacturing in the second period, the remanufacturer is responsible for collecting the end-of-life (EOL) products manufactured in the first period [51]; the decision variable here is the collection rate of the EOL products. To promote the development of remanufacturing, the government either subsidizes the remanufactured products or imposes carbon taxes on new products in both periods. Figure 1 depicts the two-period decision model. 

### 2.2. Notations

Table 1 summarizes the key symbols used in the study.

### 2.3. The Sequence of Decision Making

An OEM first determines the unit retail price of the new product and the authorization fee charged to the remanufacturer per unit. Sequentially, the remanufacturer determines the collection rate of EOL products based on the authorization fee charged by the OEM. It is assumed that the total market size is not unlimited, and the remanufactured products compete with the new products in the market, which affects the production quantity of the new products. At the same time, the collection rate affects the production quantity of remanufactured products, which further affects the sales. We solve the game with backward induction to ensure the subgame obtains perfect equilibrium conditions, which are (1) finding the perfect collection rate of EOL products by the remanufacturer; and (2) finding the solutions for the unit retail price for the new product and the unit authorization fee for the remanufactured product charged by the OEM.

### 2.4. Model Functions

(1) Demand Function

We adopt the classic function in which the demand is linearly related to the price and the number of products, which has been widely used in the literature. According to [19], the demand functions of new and remanufactured products are given as follows:

$$p_{in}=1-q_{in}-\delta q_{ir},\;p_{ir}=\delta (1-q_{in}-q_{ir})\;i\in \{N,V,S\}$$

δ represents the consumer preference for remanufactured products when compared to the new ones. Similarly, the demand function of the new products in the first period is expressed as: $p_{n}=1-q_{n}$.

(2) Collection of EOL Function

Previous studies in the field, such as [18], found that the collection cost of EOL products is a convex function of the number of collected products. Following [18], this study uses $\frac{1}{2}k{\left(\tau_{i}q_{i1}\right)}^{2}$ to represent the collection cost, where $\tau_{i}$ is the collection rate and calculated as the ratio of the number of collected EOL products in the second period and the new products sold in the first period, when the government uses action *i*, i∈{N,V,S}; k is the recycling cost coefficient of waste products.

### 2.5. Model Assumption

This study follows the previous literature to assume that EOL products not remanufactured have no economic value. The remanufacturer processes all collected EOL products and finally sells them [52]. Therefore, $q_{ir}=\tau_{i}q_{i1}$.

## 3. Model Analysis

### 3.1. Formulation and Solutions

When the government provides subsidies on remanufactured products, the revenue of the OEM $\pi_{Vn}$ is expressed as in Equation (1); and the revenue of the remanufacturer $\pi_{Vr}$ is as shown in Equation (2).

(1) $$\pi_{Vn}=(p_{V1}-c_{n})q_{V1}+(p_{Vn}-c_{n})q_{Vn}+z_{V}q_{Vr}$$

(2) $$\pi_{Vr}=(p_{Vr}-c_{r}-z_{V}+v)q_{Vr}-\frac{k}{2}q_{Vr}{}^{2}$$

When the government imposes carbon taxes on new products, the revenues of the OEM $\pi_{Sn}$ and the remanufacturer $\pi_{Sr}$ are as Equations (3) and (4), respectively.

(3) $$\pi_{Sn}=(p_{S1}-c_{n}-s)q_{S1}+(p_{Sn}-c_{n}-s)q_{Sn}+z_{S}q_{Sr}$$

(4) $$\pi_{Sr}=(p_{Sr}-c_{r}-z_{S})q_{Sr}-\frac{k}{2}q_{Sr}{}^{2}$$

To obtain the optimal solutions under the two government actions, we apply Proposition 1.

**Proposition** **1a.** *When the government provides subsidies on remanufactured products, the function of* $\pi_{Vr}$ *in Equation (2) on the collection rate* $\tau_{V}$ *is concave; The optimal solution* $\tau_{V}^{*}$ *obtained from Equation (2) can be substituted for* $q_{Vr}$ *in Equation (1), and then the revenue of the OEM* $\pi_{Vn}$ *in Equation (1) on* $q_{S1},\;q_{Sn},\;z_{S}$ *can be proven to be concave functions.*

**Proposition** **1b.** *When the government imposes carbon taxes on new products, the function of* $\pi_{Sr}$ *in Equation (4) on the collection rate* $\tau_{S}$ *is concave; The optimal solution* $\tau_{S}^{*}$ *obtained from Equation (4) can be substituted for* $q_{Sr}$ *in Equation (3), and then the revenue of the OEM* $\pi_{Sn}$ *in Equation (3) on* $q_{S1},\;q_{Sn},\;z_{S}$ *can be proven to be concave functions.*

The optimal solutions can be obtained based on Proposition 1 (see Appendix A), as shown in Conclusion 1.

**Conclusion** **1.** *The optimal solution under two different government policies is as follows. See Table 2.*

### 3.2. The Effect of Different Government Policies on the Optimal Solution

From Corollary 1, let *v* or *s* be zero; we can then obtain the optimal solutions when the government applies neither the subsidy or the carbon tax policies.

**Conclusion** **2.** The unit price and demand of new and remanufactured products under the two government policies would satisfy the following:

*(a)*  $\frac{\partial p_{V1}{}^{*}}{\partial v}=\frac{\partial p_{Vn}{}^{*}}{\partial v}=0,\;\frac{\partial p_{Vr}{}^{*}}{\partial v}<0$*;*
*(b)*  $\frac{\partial p_{S1}{}^{*}}{\partial s}=\frac{\partial p_{Sn}{}^{*}}{\partial s}>0,\;\frac{\partial p_{Sr}{}^{*}}{\partial s}>0$*;*
*(c)*  $\frac{\partial q_{V1}{}^{*}}{\partial v}=0,\;\frac{\partial q_{Vn}{}^{*}}{\partial v}<0,\;\frac{\partial q_{Vr}{}^{*}}{\partial v}>0$*;*
*(d)*  $\frac{\partial q_{S1}{}^{*}}{\partial s}<0,\;\frac{\partial q_{Sn}{}^{*}}{\partial s}<0,\;\frac{\partial q_{Sr}{}^{*}}{\partial s}>0$*.*

See Appendix A for the Proof of Conclusion 2. As also shown in [28], Conclusion 2 shows that when the government adopts the subsidy strategy, the government subsidy has the same effect on the pricing of the two products. That is, the government subsidy strategy has no impact on the retail price of the new products per unit. To obtain more government subsidies, the remanufacturer will reduce the unit price. Retail prices of remanufactured products increase sales of remanufactured products. However, when the government adopts a carbon tax policy, the original manufacturer will transfer part of the carbon tax to consumers by increasing the retail price of new products per unit. As shown in [29], due to the existence of market competition, to obtain more benefits from remanufacturing, the unit retail price of remanufactured products will be increased accordingly. Although the unit retail price of remanufactured products per unit of new products has increased, due to the existence of market competition, consumers choose to increase their purchases of remanufactured products and reduce their purchases of new products. That is, the government’s carbon tax policy reduces the sales of new products and increase the sales of remanufactured products.

**Management** **implication** **1.** *When product demand is excessive, the government should adopt a carbon tax policy. When product demand is insufficient, the government should adopt a subsidy policy. The main reason for these findings is that the government subsidy policy has no effect on the retail price or the demand of new products per unit, but increases the demand for remanufactured products.*

Corollary 1 can be obtained through the above analysis, which is as follows:

**Corollary** **1.** *The unit prices and sales volume of the two types of products under two different government policies are as follows:*

*(a)*  $p_{V1}{}^{*}=p_{Vn}{}^{*}=p_{Nn}{}^{*},\;p_{Vr}{}^{*}<p_{Nr}{}^{*}$ *;*
*(b)*  $p_{S1}{}^{*}=p_{Sn}{}^{*}>p_{N1}{}^{*},\;p_{Sr}{}^{*}>p_{Nr}{}^{*}$ *;*
*(c)*  $q_{V1}{}^{*}=q_{N1}{}^{*},\;q_{Vn}{}^{*}<q_{Nn}{}^{*},\;q_{Vr}{}^{*}>q_{Nr}{}^{*}$ *;*
*(d)*  $q_{S1}{}^{*}<q_{Nr}{}^{*},\;q_{Sn}{}^{*}<q_{Nn}{}^{*},\;q_{Sr}{}^{*}>q_{Nn}{}^{*}$

**Conclusion** **3.** *The authorization fee per unit of remanufactured products and the collection rate of EOL are as follows:*

*(a)*  $\frac{\partial z_{V}{}^{*}}{\partial v}>0,\;\frac{\partial z_{S}{}^{*}}{\partial s}=0$ *;*
*(b)*  $\frac{\partial \tau_{V}{}^{*}}{\partial v}>0,\;\frac{\partial \tau_{S}{}^{*}}{\partial s}>0$ *.*

See Appendix A for the Proof of Conclusion 3. From Conclusions 2 and 3, the OEM can take two actions to mitigate the influence of the carbon tax. One is increasing the unit price of new products to transfer the tax to consumers; another is increasing the unit authorization fee of remanufactured products to increase the total revenue from authorization. Interestingly, it is observed that the collection rate of EOL products would be increased regardless of the policy taken by the government. The main reason for this is that when the government provides subsidies on remanufactured products, it increases the sales volume of these products and inversely motivates the remanufacturer to collect more EOL products. In contrast to [29], when the government charges a carbon tax on the new products, the sales volume of new products in the first period decreases, whereas that of remanufactured products increases, in such a manner to stimulate an increase in the collection rate.

**Management** **implication** **2.** *From the perspective of remanufacturers, the government should choose a carbon tax policy. The main reason for this is that, although the government’s carbon tax policy and subsidy policy can increase the recycling rate of waste products, the government’s carbon tax policy will not change the authorization fee for remanufactured products per unit, and the government’s subsidy policy will increase the authorization fee for remanufactured products per unit.*

Based on Conclusion 3, we have Corollary 2 as follows:

**Corollary** **2.** *The relationships of the authorization fee and collection rate among the three types of products are given as:*

*(a)*  $z_{V}{}^{*}>z_{S}{}^{*}=z_{N}{}^{*}$
*(b)*  *When* $\delta <\frac{v(1-c_{n}-s)+c_{r}s}{s}$ *,* $\tau_{V}^{*}>\tau_{S}^{*}>\tau_{N}^{*}$ *, otherwise,* $\tau_{S}^{*}\geq \tau_{V}^{*}>\tau_{N}^{*}$ *.*

To better visualisation, we define $A=\frac{2(2\delta +k-\delta^{2})(1-c_{n})-\delta (c_{n}-c_{r})}{4\delta +2k-\delta^{2}}$.

**Conclusion** **4.** *The equilibrium revenue of products under two government policies satisfies this following:*

*(a)*  $\frac{\partial \pi_{Vn}{}^{*}}{\partial v}>0,\;\frac{\partial \pi_{Vr}{}^{*}}{\partial v}>0,\;\frac{\partial \pi_{Sr}{}^{*}}{\partial s}>0$ *;*
*(b)*  *When* s>A *,* $\frac{\partial \pi_{Sn}{}^{*}}{\partial s}>0$ *, otherwise* $\frac{\partial \pi_{Sn}{}^{*}}{\partial s}\leq 0$ *.*

See Appendix A for the Proof of Conclusion 4. From Conclusion 4, the government’s subsidy policy can increase the revenue of both the OEM and the remanufacturer, which is consistent with the extant studies [19,23,25]. Moreover, the carbon tax policy can increase the remanufacturer’s revenue because the unit prices and sales volumes of new and remanufactured products increase without a variation in the authorization fee per unit. By comparison, the revenue of the OEM increases as the tax per unit increases, only when the carbon tax is higher than a threshold value. The reason for this is that the OEM increases the unit price of new products to ensure that the consumers pay for a portion of the tax. This further decreases consumers intention to buy the new products and reduces the revenue of these products. At the same time, the increase in the sales of remanufactured products leads to an increase in the OEM’s total authorization fee income and offsets the decrease in income caused by the added tax. This situation can eventually increase the revenue of the OEM. Similar to the findings of [19,53], authorization enhances the position of the OEM compared to that of the remanufacturer via the imposition of an authorization fee on the remanufacturer to increase the OEM’s comprehensive revenue.

**Management** **implication** **3.** *Under the authorized remanufacturing model, when the government implements the carbon tax policy, the carbon tax quota imposed by the government regulator on the new products only increases the revenue of the original equipment manufacturer when the tax is greater than a certain threshold. However, when the tax is set at a peak level, so that the unit cost of new products is higher than that of remanufactured products, the remanufacturer will cease producing for the OEM and engage in remanufacturing.*

Based on Conclusion 4, we have Corollary 3.

**Corollary** **3.** *The relationships among the revenues of the two products under the two government policies satisfy the following:*

*(a)*  *When* s>A *,* $\pi_{Sn}{}^{*}>\pi_{Nn}{}^{*}$ *, otherwise,* $\pi_{Sn}{}^{*}\leq \pi_{Nn}{}^{*}$ *;*
*(b)*  $\pi_{Vn}{}^{*}>\pi_{Nn}{}^{*},\;\pi_{Vr}{}^{*}>\pi_{Nr}{}^{*},\;\pi_{Sr}{}^{*}>\pi_{Nr}{}^{*}$ *.*

### 3.3. Comparison of Consumer Surplus and Environmental Output

This section discusses the impacts of government policies on consumer surplus and environmental output. For better understanding, we denote $E_{N},\;E_{V}$, and $E_{S}$ as the environmental output under no policy, subsidy, and carbon tax policy, respectively, and these satisfy:

$$E_{N}=e_{n}(q_{N1}+q_{Nn})+e_{r}q_{r},\;E_{V}=e_{n}(q_{V1}+q_{Vn})+e_{r}q_{Vr},\;E_{S}=e_{n}(q_{S1}+q_{Sn})+e_{r}q_{Sr}$$

**Conclusion** **5.** *The relationships of environmental performance under the three scenarios are:*

*(a)*  *When* $\frac{e_{r}}{e_{n}}>\delta$ *,* $E_{V}>E_{N}>E_{S}$ *;*
(b) *When* $\frac{s}{v}>\frac{\delta e_{n}-e_{r}}{e_{n}(4\delta +2k-\delta^{2})-\delta e_{r}}$ *and* $\frac{e_{r}}{e_{n}}<\delta$ *,* $E_{N}>E_{V}>E_{S}$ *;*
(c) *When* $\frac{s}{v}<\frac{\delta e_{n}-e_{r}}{e_{n}(4\delta +2k-\delta^{2})-\delta e_{r}}$ *and* $\frac{e_{r}}{e_{n}}<\delta$ *,* $E_{N}>E_{S}>E_{V}$ *.*

See Appendix A for the Proof of Conclusion 5. From Conclusion 5, when the environmental output per new product is fixed and that of per remanufactured product is higher than a threshold value, the subsidy policy induces the most environmental output, and the carbon tax policy results in the least environmental output. In contrast to [19,28], when the difference between the carbon tax per new product and the subsidy per remanufactured product is larger than a threshold value, the government policies result in a lower environmental output than that of no policy, and the carbon tax policy results in the least environmental output. Finally, the environmental output is only the least under the subsidy policy when the subsidy is sufficiently high.

**Management** **implication** **4.** *A carbon tax can decrease the impact of the production processes of the two product types on the environment. Moreover, the impact of the subsidy on the environment depends on the specific product type, particularly for the remanufactured products. In addition, the subsidy only helps to decrease the environmental impact when the environmental output per remanufactured products is low. Otherwise, the subsidy may be detrimental to the environment.*

We now discuss the consumer surplus (*S_i_*) under two government policies, which satisfies:

$$S_{i}=\frac{{\left(q_{in}+\delta q_{ir}\right)}^{2}+\delta (1-\delta )q_{ir}^{2}}{2},\;i\in \{N,V,S\}$$

**Conclusion** **6.** *The relationship of the consumer surplus under the three scenarios is* $S_{V}>S_{N}>S_{S}$ *.*

See Appendix A for the Proof of Conclusion 2. From Conclusion 6, under the carbon tax policy, the OEM increases the unit price of the new product to transfer the added tax cost to consumers. In a complete market with new and remanufactured products, the remanufacturer follows the OEM and increases the product price to receive higher profit per unit, which leads to a reduction in the consumer surplus. This is similar to the findings of [26], in which the consumer surplus also decreased under the carbon tax policy. Alternatively, when a subsidy is applied to the remanufactured products, the remanufacturer decreases its product price in such a manner to increase the sales volume of the remanufactured products, leading it to increase the consumer surplus. Based on Conclusions 2, 3, and 6, we have the following implications for practitioners.

**Management** **implication** **5.** *Both the subsidy and the carbon tax are favorable to remanufacturing in authorization mode, but the consumer surplus should also be considered from the government’s perspective. Under a carbon tax policy, the OEM increases product prices to reduce the consumer surplus. Under a subsidy policy, the OEM receives the government’s subsidy by increasing the authorization fee for remanufactured products. In contrast, the unit prices of both product types decrease and the consumer surplus increases.*

## 4. Numerical Analyses

In this section, we apply the model to a well-known engine remanufacturer in China, Jinan Fuqiang Company. Compared to a new engine, a remanufactured engine consumes 50% of the economic cost and generates 60% of the environmental output [19]; that is, *C_r_* = 0.5*C_n_*, and *e_r_* = 0.4*e_n_*. From [18], *C_r_* = 0.1, *C_n_* = 0.2, *e_r_* = 0.4, *e_n_* = 1, and *k* = 1.1. The unit price of the new products and the demand for the new products in the first period under the two government policies can be easily understood, and are thus not simulated. In addition, we set the unit subsidy of remanufactured products equal to the unit carbon tax of new products, that is, *s* equals *v*, for convenience. Furthermore, in the numerical analyses, the change trend in the demand for the two products in the second cycle is consistent with the change trend in their income. Here, the change in income is selected for the analyses.

### 4.1. The Impact of the Subsidy and Consumer Surplus on the Unit Authorization Fee of Remanufactured Products

**Figure 2:**
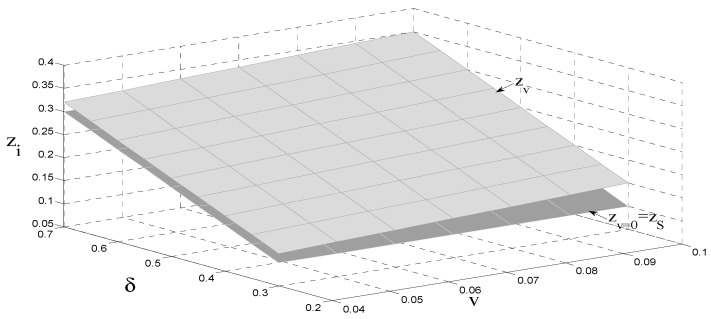
The relationship between the subsidy, consumer preference, and the authorization fee.

As shown in Figure 2, when consumer preferences are constant, the remanufactured product unit authorization fee is not related to the new product carbon tax per unit, and is positively related to the amount of government subsidies. Under authorized remanufacturing, the OEM can obtain remanufacturing revenue, and the revenue is positively correlated with the sales volume of the remanufactured products. Therefore, when the government adopts a carbon tax policy, the OEM transfers part of the carbon tax to consumers by increasing the retail price of the new products per unit. However, to further compensate for the adverse effects of the carbon tax, the OEM increases remanufacturing revenue. When the government remanufactured product unit subsidy remains unchanged, the remanufactured product unit authorization fee is proportional to the relative discount. That is, the larger the relative discount, the greater the remanufactured product unit authorization fee. The main reason for this is that the greater the consumer preference, the more enthusiastic consumers are to buy remanufactured products, and the more willing they are to pay higher prices for these products. As a result, the remanufacturer’s revenue increases. To obtain remanufacturing revenue, the OEM increases its profits by increasing the remanufacturing product unit authorization fee. Hence, we have Corollary 4 as follows.

**Corollary** **4.** *The relationship between the consumer surplus and the authorization fee per unit satisfies* $\frac{\partial z_{i}^{*}}{\partial \delta }>0$ *.*

### 4.2. The Impact of the Carbon Tax and Consumer Surplus on the Collection Rate of EOL Products

**Figure 3:**
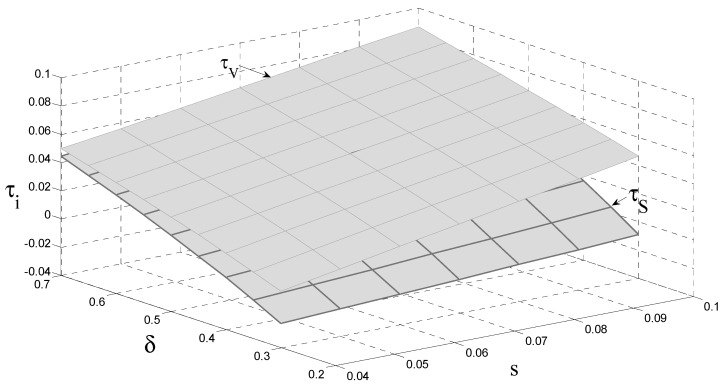
The impact of the carbon tax and consumer preference on the collection rate of EOL.

Figure 3 shows that the remanufacturer does not collect EOL products or remanufacture them when the carbon tax and consumer preference are lower than a threshold value, respectively. In addition, the remanufacturer always engages in production activities under the subsidy policy. Compared to the carbon tax policy, providing a subsidy results in a significantly larger stimulus of EOL collection. Furthermore, the collection rate of EOL is positively related to the product’s unit carbon tax and subsidy. Finally, consumer preference is always positively related to the collection rate of EOL, whether under carbon tax or subsidy policy. Hence, we have Corollary 5 as follows.

**Corollary** **5.** *The relationship between consumer preference and the collection rate of EOL products satisfies* $\frac{\partial \tau_{i}^{*}}{\partial \delta }>0$ *.*

### 4.3. The Impact of the Carbon Tax and Consumer Preference on the Unit Price of Remanufactured Products 

**Figure 4:**
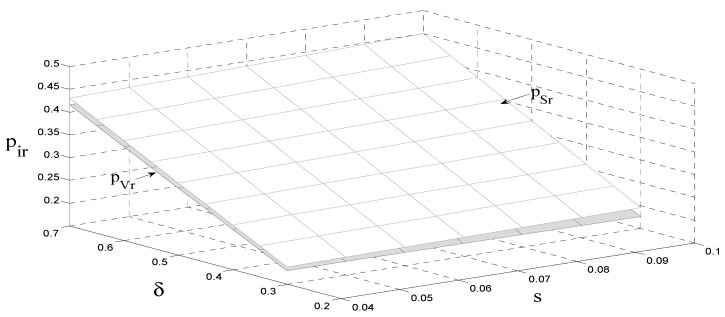
The impact of the carbon tax and consumer preference on the unit price of remanufactured products.

Figure 4 shows that the unit price of remanufactured products is positively related to the carbon tax per unit and consumer preference when there is a carbon tax charged by the government. Moreover, the unit price of remanufactured products is positively related to consumer preference under the government’s subsidy policy. The subsidy policy has a stronger effect on the unit price of the remanufactured products than the carbon tax policy. The reason for this is that the remanufacturer increases the unit price of the remanufactured products when a carbon tax is charged to increase its revenue. In addition, it decreases the unit price of the remanufactured products under a subsidy policy to expand its sale volume and further earn the subsidy. Hence, we have Corollary 6 as follows.

**Corollary** **6.** *The relationship between consumer preference and the unit price of remanufactured products satisfies* $\frac{\partial p_{i}^{*}}{\partial \delta }>0$*.*

### 4.4. The Impact of the Carbon Tax and Consumer Preference on the OEM’s Profit

**Figure 5:**
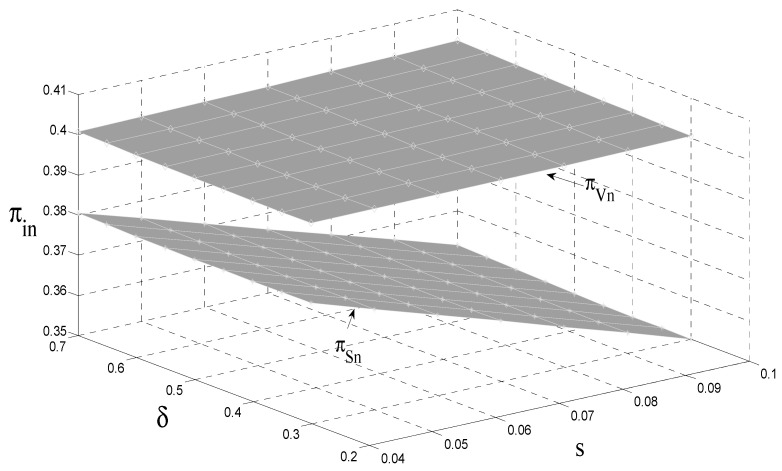
The impact of s and δ on the revenue of original manufacturers.

**Figure 6:**
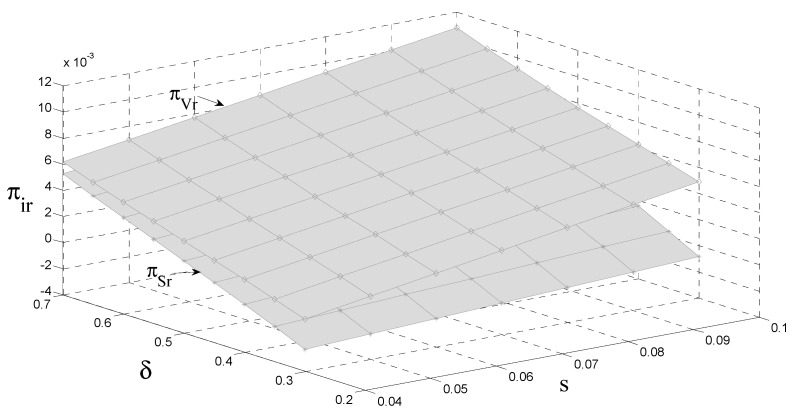
The impact of s and δ on the revenue of remanufacturers.

As can be seen from Figure 5 and Figure 6, although original manufacturers can compensate for some of the adverse effects of the carbon tax through authorized remanufacturing, in general, the government carbon tax policy has a negative impact on original manufacturers; that is, the government’s carbon tax policy reduces original manufacturing revenue. Due to market competition, when the government adopts a carbon tax policy, the original manufacturers transfer a portion of the carbon tax to consumers by increasing the retail price of a unit to reduce the impact of the tax. This indirectly reduces the sales of new products and increases the sales of remanufactured products. At the same time, the retail price of remanufactured products per unit also increases, which ultimately increases the revenue of remanufacturers. That is, the government’s carbon tax policy reduces the revenue of original manufacturers and increases remanufacturing. Compared with the government carbon tax policy, the government subsidy policy increases the income of both at the same time. However, in contrast to the government subsidy policy’s impact on the original manufacturer’s income, the government subsidy policy has an insignificant impact on the original manufacturer’s income. Due to authorized remanufacturing, the relative discount has a positive impact on the revenue of both.

### 4.5. The Impact of the Government Carbon Tax on the Original Manufacturer’s Revenue

**Figure 7:**
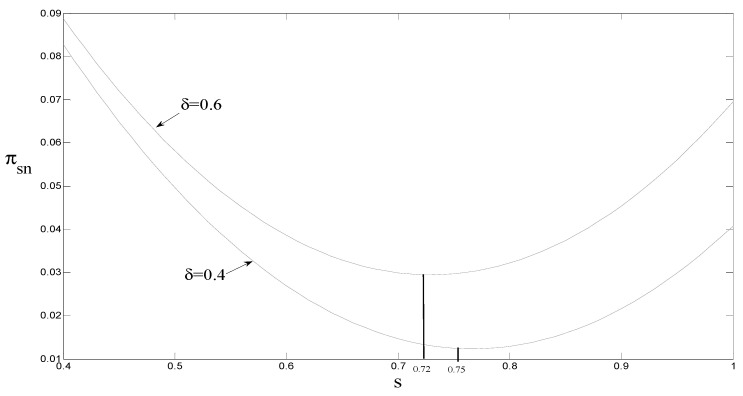
The impact of s on the revenue of original manufacturers.

It can be seen from Figure 7 that when the consumer preference is 0.6, the threshold A=0.72; that is, when the government carbon tax quota is less than 0.72, the original manufacturer’s revenue decreases as the carbon tax quota increases. Alternatively, when the government carbon tax quota is greater than 0.72, the original manufacturers’ revenues increase with the increase in carbon tax credits. When the consumer quota is 0.4, the threshold A=0.75; that is, when the government carbon tax quota is less than 0.75, the original manufacturer’s revenue decreases as the carbon tax quota increases. When the government carbon tax quota is greater than 0.75, the original manufacturer’s revenue increases with the carbon tax quota.

The main reason for these results is that when the consumer preference is 0.6 and the carbon tax amount is less than 0.72, the reduction in sales revenue of new products is greater than OEM’s remanufacturing revenue through authorized remanufacturing, and the original manufacturer’s revenue is ultimately reduced. However, when the carbon tax amount is greater than 0.72, as the carbon tax amount increases, the retail price per unit of new products also increases, reducing the sales of new products and ultimately reducing the revenue of the original manufacturer, but the sales of remanufactured products increase. The sales profit of remanufactured products increases, and the original manufacturer’s remanufacturing income through authorized remanufacturing also increases. The increase is greater than the decrease in the sales profit of new products, which ultimately increases the original manufacturer’s income.

**Management** **implications** **6.** *When consumers have low environmental awareness, the government’s carbon tax policy should be less than a certain threshold. The main reason for this is that when the government’s carbon tax amount is less than a certain threshold, the government’s carbon tax policy can not only increase the revenue of the remanufacturer, but also increase the revenue of the original manufacturer.*

## 5. Conclusions

Under authorized remanufacturing, to compare and analyze the impact of government subsidy policies and carbon tax policies on remanufacturing, firstly, a game model of manufacturing/remanufacturing under authorized remanufacturing was established. Secondly, the two types of government policy were compared and analyzed. The choice of policy has an impact on the optimal solution of the Nash equilibrium, environmental impact, and consumer surplus. Finally, to further verify the conclusions of this study, a numerical simulation was undertaken and some inferences were made. From the results of this research, the main conclusions are as follows:

(1) The government’s carbon tax policy has no effect on the authorization fee for remanufactured products per unit. When the government adopts a subsidy policy, the original manufacturer chooses to transfer part of the government subsidy by increasing the authorization fee for remanufactured products per unit to obtain remanufacturing revenue. When consumers’ environmental awareness is low, the government’s subsidy policy is more effective. The main reason for this is that, although the unit retail price of remanufactured products is lower than the unit retail price of new products, the lack of consumer awareness of environmental protection affects consumers’ enthusiasm for purchasing remanufactured products. When the government adopts a subsidy policy, it reduces the purchase cost of remanufactured products, while also increasing the consumer acceptance of remanufactured products.
(2) When the government adopts a subsidy policy, remanufacturers can increase the sales volume of remanufactured products by reducing the retail price of remanufactured products per unit to obtain more government subsidies. Under market competition, the retail price of remanufactured products per unit is reduced, which indirectly causes unit sales of new products to decrease. When the government adopts a carbon tax policy, the original manufacturer transfers the government carbon tax, which increases the retail price of new products per unit and reduces the sales volume of new products. At the same time, it indirectly increases the sales volume and the unit retail price of remanufactured products.
(3) When the consumer preference is 0.6 and the government carbon tax amount is less than 0.72, the original manufacturer’s income decreases as the carbon tax amount increases; on the contrary, the original manufacturer’s income increases as the carbon tax amount increases. The main reason for this is that when the consumer preference is 0.6 and the carbon tax amount is less than 0.72, the reduction in sales revenue of new products is greater than the original manufacturer’s remanufacturing revenue through authorized remanufacturing, and the original manufacturer’s revenue is ultimately reduced.

This article compared and analyzed the impact of government subsidies and carbon tax policies on the unit pricing, demand, revenue, and environment related to manufacturing/remanufacturing products. This research provides a basis for manufacturing/remanufacturing decisions. However, this study still has two points that can be further expanded. First, research is lacking on the government’s optimal subsidy quota and carbon tax quota in the analysis. In the future, further analysis can be undertaken of the government’s optimal subsidy and carbon tax quotas to provide a basis for the government to improve related policies. Second, consumer environmental preferences are a key factor that also affect manufacturing/remanufacturing. In the future, it will be necessary to further analyze the impact of consumer environmental preferences on manufacturing/remanufacturing.

## Figures and Tables

**Table 1:** Notation summary.

Symbol	Definition
***N***	The government uses neither subsidies nor carbon taxes
***S***	The government uses carbon taxes exclusively
***V***	The government uses subsidies exclusively
s	The carbon taxes imposed on the new product per unit
v	The subsidies on the remanufactured product per unit
*C_n_*	The production cost of new products per unit
$z_{i}$	The unit fee of authorization remanufacturing, when the government uses action policy i, i∈{S,V}
*Cr*	The unit production cost of the remanufactured product, $c_{n}>c_{r},\;s<c_{n}-c_{r}$, $v<c_{n}-c_{r}$
*p_i_*_1_	The unit retail price of the new product in the first period, when the government uses policy i, i∈{N,S,V}.
*q_i_*_1_	The sales volume of the new product in the first period, when the government uses policy i, i∈{N,S,V}.
*p_in_*, *p_ir_*	The unit retail price of the new and remanufactured product in the second period, respectively, when the government uses policy i, i∈{N,S,V}.
*q_in_*, *q_ir_*	The sales volume of new and remanufactured products in the second period, respectively, when the government uses policy i, i∈{N,S,V}.
$\pi_{in},\;\pi_{ir}$	The revenue of the OEM and remanufacturer, respectively, when the government uses policy i, i∈{N,S,V}.

**Table 2:** The optimal solution under two different government policies.

Symbol	Subsidy (i=V)	Carbon Tax (i=S)
$z_{i}^{*}$	$\frac{\delta +v-c_{r}}{2}$	$\frac{\delta -c_{r}}{2}$
$\tau_{i}^{*}$	$\frac{\delta c_{n}+v-c_{r}}{\left(2\delta +k-\delta^{2})(1-c_{n}\right)}$	$\frac{\delta (c_{n}+s)-c_{r}}{\left(2\delta +k-\delta^{2})(1-c_{n}-s\right)}$
$p_{i1}^{*}$	$\frac{1+c_{n}}{2}$	$\frac{1+c_{n}+s}{2}$
$q_{i1}^{*}$	$\frac{1-c_{n}}{2}$	$\frac{1-c_{n}-s}{2}$
$p_{in}^{*}$	$\frac{1+c_{n}}{2}$	$\frac{1+c_{n}+s}{2}$
$p_{ir}^{*}$	$\delta [\frac{1}{2}+\frac{\left(k+\delta )c_{n}+(1-\delta )(c_{r}-v\right)}{2(2\delta +k-\delta^{2})}]$	$\delta [\frac{1}{2}+\frac{(k+\delta )(c_{n}+s)+(1-\delta )c_{r}}{2(2\delta +k-\delta^{2})}]$
$q_{in}^{*}$	$\frac{1}{2}-\frac{\left(k+2\delta )c_{n}+\delta (v-c_{r}\right)}{2(2\delta +k-\delta^{2})}$	$\frac{1}{2}-\frac{(k+2\delta )(c_{n}+s)-\delta c_{r}}{2(2\delta +k-\delta^{2})}$
$q_{ir}^{*}$	$\frac{v-c_{r}+\delta c_{n}}{2(2\delta +k-\delta^{2})}$	$\frac{\delta (c_{n}+s)-c_{r}}{2(2\delta +k-\delta^{2})}$
$\pi_{in}^{*}$	$\frac{{\left(1-c_{n}\right)}^{2}}{2}+\frac{{\left(\delta c_{n}+v-c_{r}\right)}^{2}}{4(2\delta +k-\delta^{2})}$	$\frac{{\left(1-c_{n}-s\right)}^{2}}{2}+\frac{{\left[\delta (c_{n}+s)-c_{r}\right]}^{2}}{4(2\delta +k-\delta^{2})}$
$\pi_{ir}^{*}$	$\frac{(k+2\delta ){\left(\delta c_{n}+v-c_{r}\right)}^{2}}{8{\left(2\delta +k-\delta^{2}\right)}^{2}}$	$\frac{(k+2\delta ){\left[\delta (c_{n}+s)-c_{r}\right]}^{2}}{8{\left(2\delta +k-\delta^{2}\right)}^{2}}$

## Data Availability

The data was taken from the *Global E-Waste Monitoring Report 2020* and a Chinese engine remanufacturer, Jinan Fuqiang Company.

## Appendix A

**Proof.** Proof of Proposition 1. Substituting $p_{Vr}=\delta (1-q_{Vn}-q_{Vr}),q_{Vr}=\tau_{V}q_{V1}$ into Equation (2), we can rewrite $\pi_{Vr}$ as follows:

(A1) $$\pi_{Vr}=(1-q_{Vn}-\delta \tau_{Vr}q_{V1}-z_{V}+v)\tau_{Vr}q_{V1}-\frac{k}{2}{\left(\tau_{Vr}q_{V1}\right)}^{2}$$

The first-order and second-order derivatives of $\pi_{Vr}$ concerning $\tau_{V}$ are as follows:

(A2) $$\frac{\partial \pi_{Vr}}{\partial \tau_{Vr}}=(1-q_{Vn}-z_{V}+v)q_{V1}-2\delta \tau_{Vr}q_{V1}{}^{2}-k\tau_{Vr}q_{V1}{}^{2}$$

(A3) $$\frac{\partial^{2}\pi_{Vr}}{\partial \tau_{Vr}{}^{2}}=-2\delta q_{V1}{}^{2}-kq_{V1}{}^{2}<0$$

Because the second-order sufficient condition in Equation (A3) is negative, the revenue function $\pi_{Vr}$ in Equation (2) is concave in $\tau_{V}$. Let Equation (A2) equal 0, and solving simultaneously, we obtain:

(A4) $$\tau_{V}^{*}=\frac{1-q_{Vn}-z_{V}+v}{(2\delta +k)q_{V1}}$$

Substituting $p_{V1}=1-q_{V1},\;p_{Vn}=1-q_{Vn}-\delta q_{Vr},\;p_{Vr}=\delta (1-q_{Vn}-q_{Vr}),\;q_{Vr}=\tau_{V}^{*}q_{V1}$ into Equation (1), the revenue function of $\pi_{Vn}$ can be rewritten as follows:

(A5) $$\pi_{Vn}=(1-q_{V1}-c_{n})q_{V1}+(1-q_{Vn}-\delta \frac{1-q_{Vn}-z_{V}+v}{2\delta +k}-c_{n})q_{Vn}+z_{V}\frac{1-q_{Vn}-z_{V}+v}{2\delta +k}$$

The first-order and second-order derivatives of $\pi_{Vn}$ in Equation (A5) concerning *q_V_*_1_, *q_Vn_*, and *z_V_* are as follows:

$$\frac{\partial \pi_{Vn}}{\partial q_{V1}}=1-2q_{V1}-c_{n},$$

$$\frac{\partial \pi_{Vn}}{\partial q_{Vn}}=\frac{(2\delta +k)(1-c_{n})-\delta (\delta +v-c_{r})-2(2\delta +k-\delta^{2})q_{Vn}}{2\delta +k},$$

$$\frac{\partial \pi_{Vn}}{\partial z}=\frac{\delta +v-c_{r}-2z}{2\delta +k},$$

$$\frac{\partial^{2}\pi_{Vn}}{\partial q_{V1}{}^{2}}=-2,\;\frac{\partial^{2}\pi_{Vn}}{\partial q_{Vn}\partial q_{V1}}=\frac{\partial^{2}\pi_{Vn}}{\partial \tau_{V}\partial q_{V1}}=0,$$

$$\frac{\partial^{2}\pi_{Vn}}{\partial z^{2}}=-\frac{2}{2\delta +k},\;\frac{\partial^{2}\pi_{Vn}}{\partial q_{Vn}\partial z}=\frac{\partial^{2}\pi_{Vn}}{\partial q_{V1}\partial z}=0,$$

$$\frac{\partial^{2}\pi_{Vn}}{\partial q_{Vn}{}^{2}}=-2\frac{2\delta +k-\delta^{2}}{2\delta +k},\;\frac{\partial^{2}\pi_{Vn}}{\partial q_{V1}\partial q_{Vn}}=\frac{\partial^{2}\pi_{Vn}}{\partial \tau_{V}\partial q_{Vn}}=0.$$

Moreover, the Hessian matrix of $\pi_{Vn\;}$ in Equation (A5) on *q_V_*_1_, *q_Vn_*, and *z_V_* is as follows:

$$H=\left[\begin{array}{ccc} -2 & 0 & 0\\ 0 & -2\frac{2\delta +k-\delta^{2}}{2\delta +k} & 0\\ 0 & 0 & -\frac{2}{2\delta +k} \end{array}\right]$$

The first-principal, second-principal, and third-principal minors of the Hessian matrix *H* are calculated as follows:

$${\left|H\right|}_{1}=\left|-2\right|=2>0$$

$${\left|H\right|}_{2}=\left|\begin{array}{cc} -2 & \\ & -2\frac{2\delta +k-\delta^{2}}{2\delta +k} \end{array}\right|=4\frac{2\delta +k-\delta^{2}}{2\delta +k}>0$$

$$H_{3}=\left|\begin{array}{ccc} -2 & 0 & 0\\ 0 & -2\frac{2\delta +k-\delta^{2}}{2\delta +k} & 0\\ 0 & 0 & -\frac{2}{2\delta +k} \end{array}\right|=-8\frac{2\delta +k-\delta^{2}}{{\left(2\delta +k\right)}^{2}}<0$$

Therefore, Equation (A5) is concave to *q_V_*_1_, *q_Vn_*, and *z_V_*, respectively. That is, Equation (1) is concave to *q_V_*_1_, *q_Vn_*, and *z_V_*, respectively. Proposition 1b can be proven similarly. The proof of Proposition 1 is completed. □

**Proof.** Proof of Conclusion 2: We can see from Conclusion 1:

$$\frac{\partial p_{V1}{}^{*}}{\partial v}=\frac{\partial p_{Vn}{}^{*}}{\partial v}=\frac{\partial \frac{1+c_{n}}{2}}{\partial v}=0,\;\frac{\partial p_{Vr}{}^{*}}{\partial v}=-\frac{\delta (1-\delta )}{2(2\delta +k-\delta^{2})}<0$$

(a) Proven. Similarly, (b), (c) and (d) could be proved. The proof of Conclusion 2 is completed. □

**Proof.** Proof of Conclusion 3: We can see from Conclusion 1:

$$\frac{\partial z_{V}{}^{*}}{\partial v}=\frac{1}{2}>0,\;\frac{\partial z_{S}{}^{*}}{\partial s}=0$$

$$\frac{\partial \tau_{V}{}^{*}}{\partial v}=\frac{1}{\left(2\delta +k-\delta^{2})(1-c_{n}\right)}>0,\;\frac{\partial \tau_{S}{}^{*}}{\partial s}=\frac{\delta (1+c_{n})-c_{r}}{(2\delta +k-\delta^{2}){\left(1-c_{n}-s\right)}^{2}}>0$$

The proof of Conclusion 3 is completed. □

**Proof.** Proof of Conclusion 4: The proof of Conclusion 4 is similar to the proof of Conclusion 3, so no detailed proof will be given here. □

**Proof.** Proof of Conclusion 5: We can see from Conclusion 1:

$$E_{N}=e_{n}[\frac{1-c_{n}}{2}+\frac{1}{2}-\frac{(k+2\delta )c_{n}-\delta c_{r}}{2(2\delta +k-\delta^{2})}]+e_{r}\frac{v-c_{r}+\delta c_{n}}{2(2\delta +k-\delta^{2})}$$

$$E_{V}=E_{N}+v\frac{e_{r}-\delta e_{n}}{2(2\delta +k-\delta^{2})}$$

$$E_{S}=E_{N}+s\frac{\delta e_{r}-e_{n}(4\delta +2k-\delta^{2})}{2(2\delta +k-\delta^{2})}$$

When $\frac{e_{r}}{e_{n}}>\delta$, $\frac{e_{r}-\delta e_{n}}{2(2\delta +k-\delta^{2})}>0$, that is, $E_{V}-E_{N}=v\frac{e_{r}-\delta e_{n}}{2(2\delta +k-\delta^{2})}>0$ (a) Proven. Similarly, (b) and (c) can be proven. The proof of Conclusion 5 is completed. □

**Proof.** Proof of Conclusion 6: We can see from Conclusion 1:

$$S_{N}=\frac{{\left(\frac{1-c_{n}}{2}\right)}^{2}+\delta (1-\delta ){\left[\frac{\delta c_{n}-c_{r}}{2(2\delta +k-\delta^{2})}\right]}^{2}}{2}$$

$$S_{V}=\frac{{\left(\frac{1-c_{n}}{2}\right)}^{2}+\delta (1-\delta ){\left[\frac{v+\delta c_{n}-c_{r}}{2(2\delta +k-\delta^{2})}\right]}^{2}}{2}$$

$$S_{V}-S_{N}=\delta (1-\delta )\frac{{\left[\frac{v+\delta c_{n}-c_{r}}{2(2\delta +k-\delta^{2})}\right]}^{2}-{\left[\frac{\delta c_{n}-c_{r}}{2(2\delta +k-\delta^{2})}\right]}^{2}}{2}$$

$\frac{v+\delta c_{n}-c_{r}}{2(2\delta +k-\delta^{2})}-\frac{\delta c_{n}-c_{r}}{2(2\delta +k-\delta^{2})}=\frac{v}{2(2\delta +k-\delta^{2})}>0$, that is, $S_{V}>S_{N}$. Similarly, we can prove $S_{N}>S_{S}$. The proof of Conclusion 6 is completed. □
